# Discordant prenatal and postnatal RhD typing caused by a novel *RHD* frameshift variant (c.540del; p.Leu181CysfsTer48) in Swedish individuals

**DOI:** 10.1111/vox.70351

**Published:** 2026-08-20

**Authors:** Ahlam Badri, Dan Gratschev, Tove Johansson, Agneta Wikman, Tesfai Emahazion

**Affiliations:** ^1^ Department of Clinical Immunology and Transfusion Medicine Karolinska University Hospital Stockholm Sweden; ^2^ Division of Center for Hematology and Regenerative Medicine (HERM), Department of Medicine Karolinska Institutet Huddinge Sweden

**Keywords:** discordant RhD typing, frameshift deletion, next‐generation sequencing (NGS), non‐invasive prenatal testing (NIPT), RhD‐negative phenotype

## Abstract

**Background and Objectives:**

Non‐invasive prenatal testing (NIPT) for fetal *RHD* genotyping is widely used to guide anti‐D prophylaxis, but discrepancies between predicted fetal RhD status and postnatal serological typing can occur due to variant *RHD* alleles. This study aimed to confirm and characterize a suspected novel exon 4 deletion in *RHD* using multiple molecular methods and to assess inheritance.

**Materials and Methods:**

NIPT predicted an RhD‐positive fetus in an RhD‐negative pregnant woman, whereas postnatal serology showed an RhD‐negative phenotype. Molecular analysis of *RHD* was performed using the RBC‐Ready Gene CDE kit (Inno‐Train, Kronberg, Germany), targeted next‐generation sequencing (NGS) and Sanger sequencing in the family and in an unrelated RhD‐negative woman.

**Results:**

Targeted NGS identified a single‐nucleotide deletion in the *RHD* gene (c.540del), predicted to result in a frameshift and premature termination (p.Leu181CysfsTer48). Sanger sequencing confirmed the variant in the newborn and demonstrated paternal inheritance, with the father heterozygous for the deletion. The same variant was also identified in an unrelated RhD‐negative woman of Swedish origin; notably, the newborn's father was also of Swedish origin.

**Conclusion:**

This case illustrates a limitation of current NIPT approaches, which detect *RHD* sequences but not gene functionality. Molecular follow‐up is crucial in cases of discordant RhD typing.

**Highlights**

- A frameshift deletion in *RHD* exon 4 gene (c.540del; p.Leu181CysfsTer48) was identified, predicted to result in non‐functional RhD protein and RhD‐negative phenotype despite detectable *RHD* sequences.
- This case demonstrates a limitation of current non‐invasive prenatal testing assays that target selected *RHD* exons but do not assess gene functionality, leading to discordant prenatal and postnatal RhD typing with potential implications for anti‐D prophylaxis.
- Targeted next‐generation and Sanger sequencing confirmed the variant and its paternal inheritance, and the same allele was found in another RhD‐negative Swedish individual, suggesting a rare null *RHD* variant present in the Swedish population.

## INTRODUCTION

Determination of fetal RhD status is central to the management of RhD‐negative pregnancies and prevention of haemolytic disease of the fetus and newborn (HDFN). Non‐invasive prenatal testing (NIPT) using cell‐free fetal DNA has been widely implemented and fetal *RHD* screening programs significantly reduce unnecessary administration of anti‐D immunoglobulin [1, 2].

Despite high analytical performance, discordances between prenatal *RHD* genotyping and postnatal serological phenotyping are occasionally encountered [2]. These discrepancies are typically explained by variant *RHD* alleles, including weak D, partial D and null alleles arising from single nucleotide variations (SNVs), hybrid genes or structural variants [3].

Frameshift variants that introduce premature stop codons represent a well‐established mechanism leading to absence of RhD antigen expression. However, such variants may still be detected by NIPT assays targeting *RHD*‐specific exons, resulting in false prediction of RhD positivity [4].

In our setting, fetal *RHD* genotyping by NIPT is based on amplification of exon 4 only, without assessment of *RHD* gene integrity beyond this region [5].

We report a case of discordant RhD typing caused by a frameshift deletion in the *RHD* gene (c.540del; p.Leu181CysfsTer48).

## MATERIALS AND METHODS

A pregnant RhD‐negative woman underwent routine NIPT for fetal *RHD* determination. Analysis of cell‐free fetal DNA indicated the presence of *RHD*‐specific sequences, predicting an RhD‐positive fetus.

Following delivery, serological RhD typing of the newborn was performed using a standard automated microcolumn method (Quidel Ortho, Raritan, NJ, USA) and demonstrated an RhD‐negative phenotype. No weak D reactivity was observed. Due to the discrepancy between prenatal and postnatal results, molecular analysis of the *RHD* gene was initiated.

Informed consent was not obtained, as this is a methodological report based on fully anonymized data and no identifiable participant information is disclosed.

### Serological testing

RhD typing was performed using BioVue cassettes with licensed anti‐D reagents in an automated system (Quidel Ortho), according to standard laboratory protocols.

### Sequence‐specific primer‐PCR

Genomic DNA from the newborn was analysed using targeted RBC‐Ready Gene CDE kit (Inno‐Train, Kronberg, Germany): This method leverages sequence‐specific primer (SSP) for DNA amplification, allowing detection of specific genetic markers on the FluoVista instrument, following the manufacturer's protocols.

### Next‐generation sequencing

Genomic DNA from the newborn was analysed using targeted next‐generation sequencing (NGS) covering the *RHD* gene [6]. Sequence alignment and variant calling were performed against the reference genome (GRCh38). Variants were annotated using human genome variation society nomenclature.

### PCR amplification

PCR amplifications were performed in a 20 μL final volume containing 2 μL of template DNA (10 ng/μL), 10 μL AmpliTaq 360 Gold PCR Master Mix (ThermoFisher), and 8 μL (0.5 μM) of each M13 tagged primer, forward 5′‐GTAAAACGACGGCCAGGGACTATCAGGGCTTGCCC‐3′ and reverse 5′‐CAGGAAACAGCTATGACACTTGGTAAAGGAGGGAGATTT‐3′. The reaction mixtures were preheated to 94°C for 7 min, followed by 34 amplification cycles (94°C for 30 s, 60°C for 35 s and 72°C for 45 s), with a final extension at 72°C for 45 s.

### Sanger sequencing

The identified variant was analysed by bidirectional Sanger sequencing in the newborn and both parents. PCR amplification targeted exon 4 of the *RHD* gene, encompassing the deletion site, using the same primer pair as in the initial analysis. The sequence data were analysed by Seqscape4 software (Applied Biosystems, Foster City, CA, USA).

## RESULTS

SSP‐PCR analysis demonstrated the presence of the *RHD* gene but indicated an *RHD* variant that could not be classified in the newborn. Subsequently, NGS identified a heterozygous single‐nucleotide deletion in exon 4 of the *RHD* gene, c.540del. This variant is predicted to cause a frameshift leading to a premature termination codon (p.Leu181CysfsTer48). The resulting truncated protein is expected to lack essential transmembrane domains, leading to absence of RhD antigen expression on red blood cells.

Sanger sequencing confirmed the presence of the c.540del variant in the newborn (Figure S1). Parental testing demonstrated that the father was heterozygous for the same deletion, while the mother did not carry the variant (Figure 1), consistent with paternal inheritance. Beyond this index case, the same c.540del variant has been identified in another RhD‐negative woman in the Swedish population. Notably, both the newborn's father and this woman originate from the Swedish population.

**FIGURE 1:**
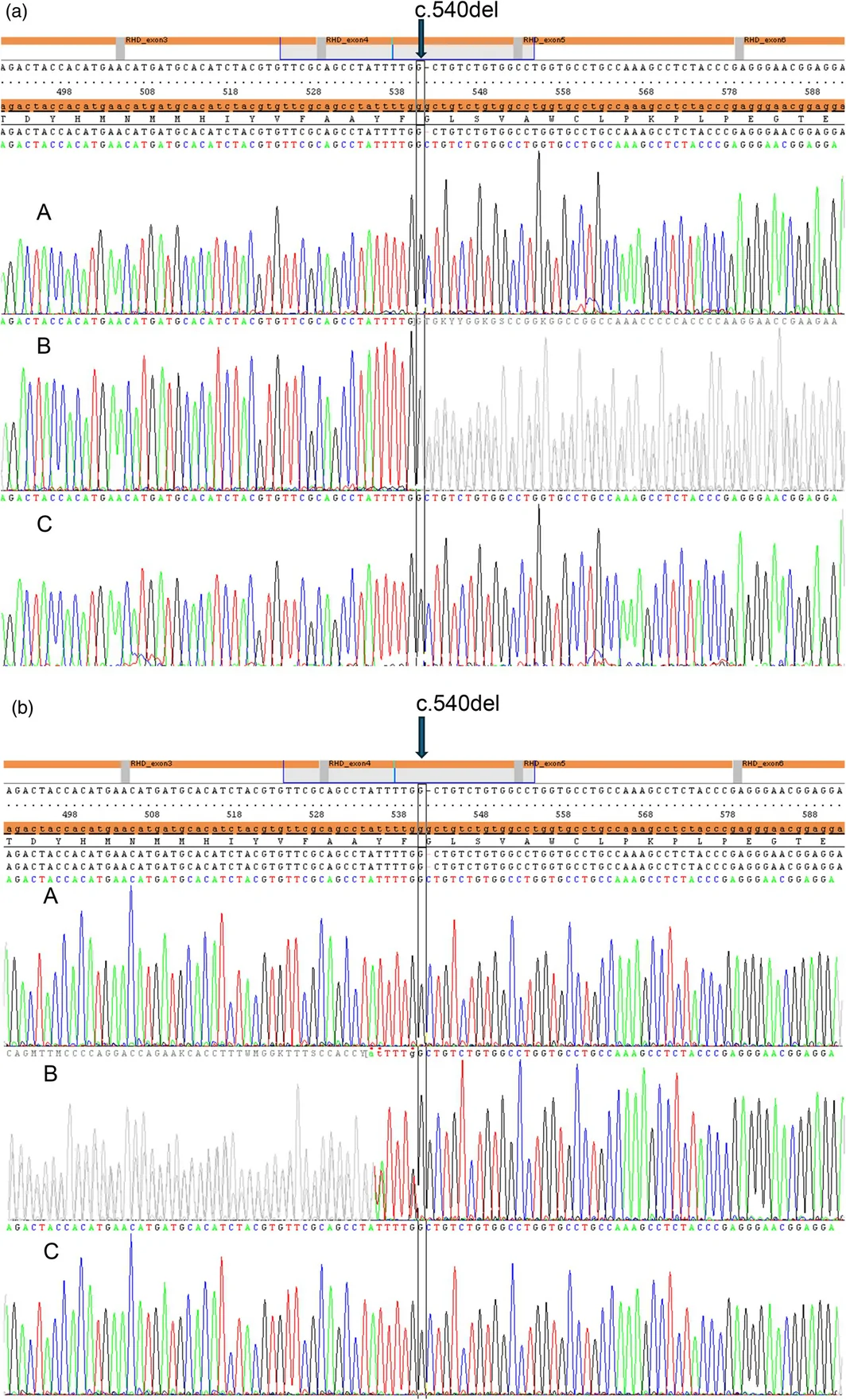
Sanger sequencing alignment confirming the variant in the proband, heterozygosity for the same deletion in the father and the presence of the same deletion in another unrelated RhD‐negative woman. The deletion site is indicated by an arrow. (a) Sanger sequencing alignment obtained using the forward primer. (b) Sanger sequencing alignment obtained using the reverse primer. A: Proband, B: father, C: unrelated RhD‐negative woman.

To ascertain whether this represented a known or a novel variant, we performed a systematic search of the International Society of Blood Transfusion (ISBT) Blood Group Database (https://blooddatabase.isbtweb.org; accessed 26 May 2026), alongside a comprehensive review of relevant peer‐reviewed publications. In the absence of any corresponding previously described variant, we classified and report this as a novel *RHD* variant.

## DISCUSSION

This case illustrates a clinically relevant discrepancy between prenatal *RHD* genotyping and postnatal serological RhD typing. The NIPT assay detected the presence of *RHD* sequences in fetal DNA but did not distinguish between functional and non‐functional alleles.

The identified variant, c.540del (p.Leu181CysfsTer48), introduces a frameshift and premature stop codon, likely resulting in nonsense‐mediated decay or production of a truncated, non‐functional protein. This explains the absence of detectable RhD antigen despite the presence of *RHD* gene sequences.

Such null alleles represent a known limitation of NIPT assays that rely on amplification of selected *RHD* exons. Detection of gene fragments does not guarantee expression of a functional RhD protein. Consequently, rare variants can lead to false‐positive predictions of fetal RhD status.

From a clinical perspective, these discrepancies are important because they may influence decisions regarding anti‐D prophylaxis. While NIPT remains highly reliable at the population level, this case underscores the need for confirmatory testing when discordant results arise.

The choice of assay targets and assay design is therefore critical. In our setting, the NIPT assay includes *RHD* exon 4 and will detect the presence of this exon in the c.540del allele. However, the assay is designed to indicate the presence of a selected *RHD* exon rather than to elucidate its exact sequence composition and therefore does not identify the underlying frameshift variant or classify the allele as null. Likewise, an assay targeting only *RHD* exons 5 and 7 would be expected to yield a positive *RHD* result in this case, while remaining blind to the disruptive change in exon 4. This illustrates that exon‐based presence/absence assays, even when multiple exons are targeted, may still produce false‐positive *RHD* calls in the presence of truncating or nonsense variants that are not specifically interrogated at the sequence level.

Furthermore, this case illustrates how NGS and Sanger sequencing provide complementary, independent lines of evidence that strengthen the characterization of *RHD* variants and their inheritance patterns. Beyond the index case, the same c.540del variant was also identified in an unrelated RhD‐negative woman; notably, both the newborn's father and this woman originate from the Swedish population. Identification of such variants in more than one RhD‐negative individual of Swedish origin suggests that this allele may, although likely rare, be present in the Swedish population and contributes to improved understanding of *RHD* molecular diversity and supports refinement of prenatal testing strategies.

## CONCLUSION

We describe a frameshift deletion in the *RHD* gene (c.540del; p.Leu181CysfsTer48) resulting in a non‐functional RhD protein and discordant prenatal and postnatal RhD typing. This case emphasizes the limitations of current NIPT approaches, which detect *RHD* sequences but do not evaluate gene functionality, and highlights the importance of molecular follow‐up in discrepant cases.

## CONFLICT OF INTEREST STATEMENT

The authors declare no conflicts of interest.

## Supporting information


**Figure S1.** Agarose gel electrophoresis image of the PCR product spanning exon 4, which was subsequently subjected to Sanger sequencing. Lanes 1–6 correspond to: (1) size marker (100–500 bp DNA Ladder, Olerup SSP), (2) negative control, (3) proband, (4) mother, (5) father and (6) unrelated RhD‐negative woman. PCR products were separated on a 1% agarose gel, stained with GelRed (BioNuclear) and visualized under UV illumination.

## Data Availability

The data that support the findings of this study are available on request from the corresponding author.
