# Supplementary material for: Discordant prenatal and postnatal RhD typing caused by a novel RHD frameshift variant (c.540del; p.Leu181CysfsTer48) in Swedish individuals
Source: Vox Sang. 2026 Aug 20;121(11):1772–6. doi: 10.1111/vox.70351 (PMC13748418; doi:10.1111/vox.70351)
Supplement: Supplementary file 1 [file VOX-121-1772-s001.docx]

Supplementary figure. Agarose gel electrophoresis image of the PCR product spanning exon 4, which was subsequently subjected to Sanger sequencing.

1	2	3	4	5	6

Lanes 1-6 corresponds to: (1) size marker (100-500 bp

DNA Ladder, Olerup SSP), (2) negative control, (3) proband, (4) mother, (5) father, and (6) unrelated RhD-negative woman. PCR products were separated on a 1% agarose gel, stained with GelRed (BioNuclear), and visualized under UV illumination.
